# Impacts of the Invasive *Impatiens glandulifera*: Lessons Learned from One of Europe’s Top Invasive Species

**DOI:** 10.3390/biology10070619

**Published:** 2021-07-03

**Authors:** Stephanie Coakley, Carloalberto Petti

**Affiliations:** Department of Science and Health, EnviroCORE, Institute of Technology Carlow, R93 V960 Carlow, Ireland; stephanie.coakley@Itcarlow.ie

**Keywords:** invasive, *Impatiens glandulifera*, conservation biology, alien species, biological invasion

## Abstract

**Simple Summary:**

Plants and animals are a part of a larger system, commonly referred to as an ecosystem. This generally implies a balance existing between prey and predators. The unintentional introduction of a species in a new environment can lead to a significant alteration of the ecosystem(s) and the uncontrolled spread of the species. When this takes place, the introduced species is referred to as invasive. Invasives can affect the ecosystem in profound ways, and generally, negatively impacting on the native species. This manuscript reviewed the current knowledge of one of Europe’s top invasives, the Himalayan balsam (*Impatiens glandulifera*). It provides insights on the species and what have we learned from this invasive species.

**Abstract:**

Biological invasions are renowned for their negative ecological and economic implications, however from studying invasions invaluable insights can be gained in the fields of ecology and evolution- potentially contributing towards conservation plans to deal, not only with biological invasion, but with other concerning issues, such as climate change. *Impatiens glandulifera*, or Himalayan balsam, is widely considered to be a highly problematic invasive, having spread across more than thirty countries during the past century. This paper will examine the findings which have arose from studying *I. glandulifera* and its impacts on the invaded ecosystem.

## 1. Introduction

**Figure 1:**
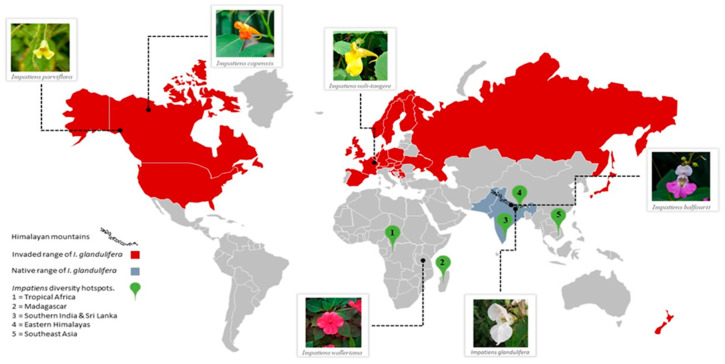
The distribution range of *I. glandulifera* in its native range (blue) and invaded range (red). The broad distribution of *I. glandulifera* enables it to be studied in a multitude of different habitats across a broad latitudinal range, highlighting the effect of an invasive across a geographically diverse area. Furthermore, these insights may help develop assisted migration conservation plans. The genus *Impatiens* is often referred to as ‘poor man’s orchids’ or ‘jewel weeds’- a reference to the high level of phenotypic diversity boosted by the circa 1000 species; among these, there is a high level of endemism, particularly in the five diversity hotspots. *Impatiens noli-tangere* is the only species native to Europe and is often used in comparison studies [12,13,14,15]. *Impatiens walleriana* and *Impatiens parviflora* are both considered invasive, with *Impatiens capensis*, *Impatiens balfourii* and *Impatiens edgeworthii* (the latter two found nearby *I. glandulifera* in the Himalayas) considered potentially invasive [16]. Sources of CreativeCommons images: *I. balfourii* [17] CC by-SA 4.0, *I. capensis* [18] CC by-NC-SA 2.0, *I. edgeworthii* [19] CC by-SA-4.0, *I. parviflora* [20] CC by 2.0, *I. noli-tangere* [21] CC by-SA-2.0, *I. walleriana* [22] CC by-SA 4.0.

Invasive species are the second greatest threat to biodiversity, capable of altering the functioning of ecosystems and the provision of ecosystem services [1]. However, biological invasions can also be viewed as “unintended large-scale experiments in nature”, resulting in many valuable insights to ecology and evolution [2,3,4]. Considering the extend of the spread of *I. glandulifera* since its introduction to Europe from the Himalayas in 1839 (Figure 1), there is an abundance of lessons which can be learned from studying the invasion of *I. glandulifera* [5,6]. *Impatiens glandulifera* was listed as an invasive alien species of Union concern in the 2017 update to Regulation (EU) No. 1143/2014 [7,8]. Understanding both the ecology of an invasive species, such as *I. glandulifera* and, the full range of its effects on the invaded ecosystem is essential in the formulation of effective control strategies and rehabilitation of invaded habitats [4,9,10,11].

## 2. Lesson Number One: *Impatiens glandulifera* Has a Differential Effect on Invaded Ecosystems

The biotic resistance hypothesis asserts that communities which are more diverse are less susceptible to biological invasions than less diverse communities [23,24]. The riparian habitats dominated by *I. glandulifera* are prone to invasives, in part due to their high disturbance regimes [25].

Research has revealed that the soil microbiome of coniferous forests is more sensitive to the influence of invasion by *I. glandulifera* than that of deciduous forests, which may consequently lead to a stronger impact of *I. glandulifera* [26]. Furthermore, open habitats (such as riverbanks and floodplains) tend to experience more pronounced effects than shaded habitats, with *I. glandulifera* exerting the strongest effect on species within habitats which are bright and very wet [27]. Interestingly, Čuda et al. [28] asserted that the impacts of this invasive are comparable in both riparian and forested habitats, with the extent of the impact depending on the dominance and residence time of *I. glandulifera* in the invaded community.

Contrary to reports that *Impatiens glandulifera* can significantly reduce species richness and diversity (by ~11 to 30 %), there are reports of this invasive having no/negligible impacts on native flora [29,30,31,32,33]. As aforementioned, the environmental requirements of the flora can determine the extent to which *I. glandulifera* can impact upon them. Light dependent species are more significantly impacted by invasives such as *I. glandulifera*, whose towering structures create canopies which can impede light filtering through to smaller plants; research has shown that the manual removal of *I. glandulifera* is likely to favour select plant species, for example species which are less-shade tolerant are likely to benefit from the removal of *I. glandulifera*. Whereas other species, such as those which are less drought-tolerant, will likely be eliminated following removal of *I. glandulifera* [10,34,35]. 

Although *I. glandulifera* is seen to alter the floristic composition of the invaded habitat to varying degrees [11,26,32,36], it is important to note that environmental factors (such as water availability) can influence species composition- indeed environmental conditions have been observed to have a stronger impact on native vegetation than on *I. glandulifera* [27,29]. Furthermore, seasonal variations over a few years (particularly for annual plants) ought to be taken into account, as evaluating impacts based on a single season can result in biased or misleading results [32,37]. Contrary to other dominant invasive plants, *I. glandulifera* tends to suppress, rather than eradicate, species from the invaded region. Encouragingly, *I. glandulifera*, which most commonly impacts widespread dominant ruderal species, does not appear to displace rare species of reed communities/floodplains or woodlands [9,11,27,29,34].

However, it is important to highlight that the aforementioned studies investigating the impact of *I. glandulifera* on phytodiversity were conducted in various geographic locations (i.e., Germany [29], England [34], Czech Republic [32,36] and Switzerland [31]), in a number of different types of habitats (both riparian and forest habitats- and consequently different environmental conditions) and different study designs were utilized (sampling of invaded and adjacent non-invaded areas versus the inclusion of ‘removal plots’); these varied environmental conditions and study designs can all influence the extent of impact of the invasive species- further highlighting the importance of conducting impact studies for specific habitats prior to developing management strategies.

### Variable Impact of I. glandulifera on Invertebrate Communities

*Impatiens glandulifera* was observed to have a differential effect on invertebrates, where aboveground species are typically more sensitive to invasion- exemplified by the overall decrease in their abundance and diversity [10,11,38]. 

**Figure 2:**
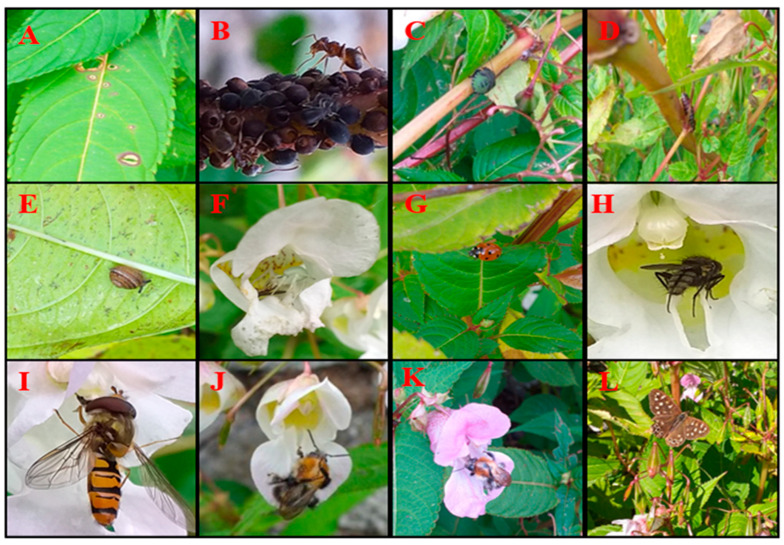
Invertebrate communities associated with *I. glandulifera* are said to be impoverished and though *I. glandulifera* is considered to be free of natural enemies which constrain its growth in the invaded range, it is not devoid of visitors [42]. Herbivory can be observed on the leaves (**A**), both pollinators and phytophagous insects (see *Aphis fabae* (**B**), *Palomena prasine* (**C**) and Dermaptera (**D**)) are observed to visit *I. glandulifera*. Gastropods (**E**) of all sizes can be observed on the stems, leaves and even inside the flowers of *I. glandulifera*. Multiple species of spiders can be observed casting webs between flowering stems, or even found, as is the case for *Misumena vatia*, residing inside its flowers (**F**). In addition to *Palomena prasine*, other beetles, such as *Coccinella septempunctata* (**G**), visit *I. glandulifera* as well as various flies, including Sarcophagidae species (**H**). Many hoverfly species, including *Episyrphus balteatus* (**I**), can be seen feeding on the pollen of *I. glandulifera*, while bumblebee species prefer the rich nectar of this invasive. Pollinators can often be seen grooming copious amounts of pollen off themselves upon leaving the flowers of *I. glandulifera* (**J**). Multiple *Bombus* species visit the flowers of this invasive- including *Bombus hypnorum* (**K**). Several butterfly species also visit this invasive- including *Pararge aegeria* (**L**).

*Impatiens glandulifera* is typically considered to be unpalatable to invertebrates and monotypic stands are of little nutritional value to native invertebrates; this is supported by evidence of decreased abundance of aboveground invertebrate populations in invaded plots and an increase in their abundance following the removal of *I. glandulifera* [10,11]. However, *I. glandulifera* is not devoid of herbivorous invertebrates (Figure 2), with species such as *Aphis fabae* (Hemiptera), *Deilephila elpenor* (Lepidoptera) and Gastropoda, known to feed on this invasive [5,39]. 

Furthermore, the same groups of herbivores, predators and detritivores which were observed to be negatively affected in aboveground populations, were found to be largely unaffected- or even positively affected- in belowground populations [10,11,40,41]. Indeed, gastropods and two of the most abundant microarthropods in leaf litter/soil (Collembola and Acari), which play a key role in nutrient cycling, are positively associated with *I. glandulifera* [11,41]. Interestingly, research has also shown that where *I. glandulifera* is associated with a more heterogeneous fauna, this is at the expense of invertebrate diversity and abundance overall- though it is important to note that more morphospecies are indicative of sites invaded by *I. glandulifera* than fellow invasive plant *Fallopia japonica*, suggesting that *F. japonica* is more prohibitive to invertebrates than *I. glandulifera* [38].

## 3. Lesson Number Two: Invasive Plants Can Have “Beneficial Uses”

Based primarily on what is conceived to be a low threat to phytodiversity, several authors have suggested that *I. glandulifera* does not pose a significant threat to invaded habitats as some other invasive plant species. As an annual with fluctuating populations, *I. glandulifera* is not able to dominate habitats for entire growing seasons in the same way as rhizomatous perennials such as *Fallopia* spp. or *Solidago* spp. [29,30,36]. Therefore, it has been proposed that control efforts should focus on invasive species which exert stronger effects, such as *Fallopia* spp., except in areas which host rare species [27,29,34,36]. Furthermore, there is an argument to allow some populations of invasives to persist because of some of their beneficial features. In fact, similar to its congener *Impatiens walleriana*, *I. glandulifera* has shown promise as a potential hyperaccumulator of cadmium [43,44,45]. Invasive plants offer a promising option for phytoremediation and their use in remediating our environment could offer a sustainable management option for this problematic plants. Moreover, there is the opportunity to utilise many invasive plants, including *I. glandulifera*, as a viable feedstock for bioenergy production [46]. Furthermore, two glucosylated steroids (glanduliferins A and B) isolated from *I. glandulifera* have shown anti-cancer potential against hepatocellular carcinoma cells in in vitro studies [47]. More recently, an antidepressant-like effect has been observed from hyperoside and protocatechuic acid which were both isolated from *I. glandulifera* [48].

### Impatiens glandulifera and Pollinators

It is important to note that one might expect even the most damaging invasive species to experience some beneficial relationships- especially considering the number of ornamental species (including *I. glandulifera*) which have become invasive [5,49,50]. Beneficial impacts on specific tropic groups could therefore be considered an extension of the differential impacts of the invasive species. However, in developing management plans for invasive species all relationships and impacts ought to be considered-both positive and negative. For instance, the 2019 update to Regulation (EU) No. 1143/2014 notes that measures must be taken to aid the recovery of ecosystems following the removal of the invasive [51], thus, if pollinators were dependent on the invasive species, then an alternative resource ought to be provided following invasive removal to sustain these pollinators.

*Impatiens glandulifera* is highly attractive to pollinators, rapidly developing highly efficacious relationships with pollinators in invaded regions, facilitating its establishment and spread [52,53,54]. The high rate of production of large volumes of nutritious nectar is largely responsible for preferentially attracting pollinators [52]. While there is certainly evidence to support the preferential attraction of pollinators away from nearby native species [39,52,55], this is not always correlated with a negative impact on native flora. An increase in species richness, visitor abundance and flower visitation was reported for plots invaded by *I. glandulifera* in multiple studies, resulting in a facilitated increase in pollinator visits for native species [55,56,57]. As seen with the indigenous *Lythrum salicaria* L., when there is an overlap in the pollinator community, competition with *I. glandulifera* can occur [55]. However, despite experiencing a substantially higher visitation rate of pollinators than native plants, *I. glandulifera* appears to increase the attractiveness of the collective set of flora to key pollinators. In fact, the total amount of native pollen being transferred and the size of the resulting seed set was observed to remain similar in invaded and non-invaded regions for several native plant species [55,56,57,58]. 

## 4. Lesson Number Three: *Impatiens glandulifera* Is a Transformer 

*Impatiens glandulifera* is considered a transformer, an invasive which can change the characteristics or conditions of an ecosystem over a large area of space [59,60,61]. However, the influence of invasion on ecosystems can vary considerably, with *I. glandulifera* presenting “marginal effects on vegetation and soil properties”-differentiating it from other major invasive transformer species [61]. Invasive plants can indirectly modify abiotic habitat conditions such as physical and chemical soil properties (e.g., soil moisture, pH, nutrients) [62,63]. These are important factors in determining the survival of the soil microbiome, seeds within the soil seed bank and other plants [26,64]. There is evidence to suggest that *I. glandulifera* can alter or degrade ecosystem services, such as erosion control, pollination and nutrient cycling [65,66]. Bieberich [33] proposed that *I. glandulifera* is a “back-seat driver” of change with ecological changes (such as disturbances, alteration in nutrients, pollution) facilitating its invasion, paving the way for it to become a driver of future change; this may help to explain the differential impact of *I. glandulifera*, it may be that the stability of the ecosystem can influence the extent of its impacts more so than the habitat type. The microclimate of the soil (temperature, humidity and aeration) can be altered by the shading effects of plants [67]. *Impatiens glandulifera* has been observed to increase soil pH and moisture, which can indirectly affect plant, fungal and bacterial communities [11,26,40,41]. Furthermore, it has been suggested that the generation of a more favourable habitat during the dry summer period, by means of altering the soil microclimate and nutrition, is responsible for the increase in belowground invertebrate communities; indeed, the total abundance of Collembola was found to be 569 % higher in invaded plots [11,40].

### Impatiens glandulifera Is Associated with the Erosion of Riverbanks

There is some evidence to suggest an increase in the risk of flooding following the autumn dieback of large stands of *I. glandulifera* on riverbank which destabilises the riverbank, promoting soil erosion along invaded riparian zones [68,69,70]. The subsequent incorporation of dead plant material into water bodies can increase the risk of eutrophication [68]. *Impatiens glandulifera* can increase the amount of organic matter entering the ecosystem, but some of this (such as the stems) has a slower rate of decomposition [11]. An alteration in soil nutrients has been observed in plots invaded by *I. glandulifera* in several studies; higher levels of phosphate [9,40,71,72], magnesium and potassium [72]. However, a lower level of potassium was also noted in plots invaded by *I. glandulifera*; the observed higher uptake of potassium was believed to enable *I. glandulifera* to attain large heights under low irradiance. This contrasting impact on potassium levels exemplifies the aforementioned ecosystem dependent effects of *I. glandulifera* [29].

## 5. Lesson Number Four: The Microbiome Plays an Important Role in the Management of Invasive Species

### 5.1. Impatiens glandulifera and the Soil Microbiome

Most studies report a negative effect of *I. glandulifera* on soil fungal communities (see [71,73]). Interestingly, in a study completed in deciduous and coniferous forests, plots invaded by *I. glandulifera* were characterised by a higher diversity and altered composition of soil fungal communities, with the effect being most prominent in coniferous forests [26]. This increase in soil fungal richness was proposed to be the result of an increase in saprophytic fungi, supported by a 37% lower leaf litter biomass- indicative of higher activity of decomposers [26]. A highly varied arbuscular mycorrhizal fungi (AMF) colonisation rate of 10 to 90% is reported for *I. glandulifera*, suggesting both that this invasive is not strictly dependent on mycorrhizal symbiosis and that it does not experience the same level of beneficial symbiosis with AMF as it does in the native zone; this may be due to a lack of specificity between this non-native plant and the AMF it encounters in various habitats within the invaded range [26,71,74,75,76,77]. In the invaded range, *I. glandulifera* appears to have a low threshold of AMF colonisation, above which does not result in increased vegetative growth. Indeed, the level of AMF colonisation is halved (22.8% ± 3.69 versus 44.6% ± 1.91 *p* < 0.01) when *I. glandulifera* is grown in previously invaded (‘conditioned’) soil [71]. Furthermore, studies have shown how invasion by *I. glandulifera* reduces mycorrhizal colonisation of forest plants, thereby reducing the survival of tree saplings [9,71,73].

There are several mechanisms which may be responsible for the influence of *I. glandulifera* on the soil microbiome. For example, depleted fungal communities could be the result of fungi being starved of its plant-acquired carbon if it cannot form a mutualism with *I. glandulifera* and if, because of *I. glandulifera*’s dominance, there are no other plant species present for it to engage with. It has also been proposed that *I. glandulifera* selectively eliminates mycorrhizal fungi which are beneficial to native plants [9]. Alternatively, the impact of *I. glandulifera* may be an indirect effect of the induced alteration in soil properties and the release of allelopathic compounds into the soil [4,73,78,79].

### 5.2. Endophytes Interact with and Interfere with Biological Control

*Impatiens glandulifera* partially conforms to the endophyte-enemy release hypothesis- it is an introduced species with an impoverished endophyte complement (low diversity), acquired from the local environment, i.e., an increase in dissimilarity was observed with increasing distance between populations. Nevertheless, three endophytes (*Colletotrichum acutatum, Alternaria alternara* and *Cladosporium oxysporum*) were common and appeared to be antagonistic to the rust fungus *Puccinia komarovii var. glanduliferae* var. nov., a biological control agent released in the UK in 2014, with a second strain being released in 2017 [80,81,82,83]. The presence of the aforementioned fungi, singularly or in combination, reduced the detrimental effect of the rust on plant biomass under glasshouse conditions; this could help to explain the resistance of several populations of *I. glandulifera* across the UK and Wales, where the establishment of the rust has been described as patchy [82,83].

## 6. Lesson Number Five: The Importance of Field Experiments

### 6.1. Allelopathy

The novel weapon hypothesis is believed to apply to *I. glandulifera*, acting to increase its success as an invasive [8,77]. According to this hypothesis, the production of 2-methoxy-1,4-naphthoquinone (2-MNQ), a potential allelochemical, could provide a competitive advantage to *I. glandulifera* by inhibiting germination or the growth of plant or disrupting symbiotic associations between plants and the soil microbiota [14,72]. While in vitro studies have provided support for this hypothesis, aqueous extracts have shown strong inhibitory effects on a variety of plant and fungal species, with the allelopathic effects of *I. glandulifera* on *Dactylis glomerate* stronger than that of native forbs and other invasives, such as *Fallopia japonica* and *Solidago gigantea*, the results of a field trial experiment have suggested that the allelopathic effects of *I. glandulifera* (and other invasives) do not suppress germination more than a native plant community- indeed, more seeds germination from the seedbank on invaded soil than non-invaded soil [73,84,85,86].

### 6.2. Delayed Response of Ecosystems to Invasion

Field trials have revealed that even though *I. glandulifera* does not appear to significantly reduce phytodiversity in the short term, the long-term effects of invasion can be more substantial; after circa ten years of repeated invasion by *I. glandulifera*, reductions of 25% in the diversity of aboveground flora and 30% in the diversity of the soil seedbank were observed [31]. Invasives which impact the seedbank can affect future generations of plants, even after the invasive has been removed from the environment as phytodiversity recovery is dependent on recruitment from the seed bank, seed rain and vegetative expansion [34,87]. However, it is worth noting that populations of *I. glandulifera* fluctuate annually; for example, flooding can strongly shape populations by aiding dispersal but inhibiting germination/growth- depending on the timing of the flooding event [27,38,88,89]. Therefore, the observed potential long-term impacts of invasion may be more applicable to inland habitats, such as the woodlands which are increasingly being invaded by *I. glandulifera*- the negative impacts on native vegetation in foothills of Tatra mountains support this premise [35,90].

## 7. Lesson Number Six: Insights into Considerations for Conservation Efforts

### 7.1. Promotion of Native Species May Inhibit Invasion

Just as disturbance events promotes invasion, habitats which host a higher level of phytodiversity are more resilient against invasive species- in particular, a high abundance of native dominant plants appears to increase the resistance of the habitat to invasion [25,91,92]. A recent study has argued that higher phytodiversity does not provide resistance to invasion by *I. glandulifera* but rather the identity of natives in the resident community may be key to limiting its success, as *Urtica dioica* was the only tested species which was found to reduce the growth of *I. glandulifera* [93]. It has been suggested that management efforts should focus on promoting environmental conditions which favour native vegetation; for example, regulating river flow to maintain high spring water levels could promote native dominants while impeding the germination of *I. glandulifera*- furthermore, targeting *I. glandulifera* in the spring could prevent the formation of large summer monocultures [25].

### 7.2. The Mechanism of Control Needs to Be Carefully Reviewed for Its Potential Impacts on the Ecosystem

Another important conservation consideration is that the mode of controlling the invasive also affects other species which inhabit the invaded environment. This is exemplified by the response of invertebrates to manual and biological control of *I. glandulifera*. Despite a more significant increase being observed for Coleoptera and Gastropoda when manual methods of controlling *I. glandulifera* were employed, a decrease in the total abundance of belowground invertebrates, which are important to key ecological processes such as nutrient cycling, was also observed [10]. Biological control, using the rust *P. glanduliferae*, not only offers a natural and sustainable means of self-regulating invasive populations, but it may also offer a gentler means of aiding habitat recovering over time. Using *P. glanduliferae* was shown to control the growth of *I. glandulifera* without negative impacts on the environment, indeed, an increase in the total abundance of both above- and belowground invertebrates [10].

### 7.3. The Microbiome May Be Key to the Successful Rehabilitation of Damaged Environments

Furthermore, if the microbiome has been negatively impacted by *I. glandulifera*, it too may need to be assisted to aid the recovery of the invaded environment, as the soil microbiome plays a key role in the functioning of ecosystems and enhances the fitness and survival of plants with which it forms mutualisms [9]. Indeed, fungi have been successfully applied to the restoration of degraded environments [94]. A study investigating the use of a commercial mycorrhizal inoculum during *I. glandulifera* different control treatments resulted in some interesting revelations. It was suggested that the rust altered the effect of *I. glandulifera* on the soil microbiome, potentially lessening the impact of *I. glandulifera* on AMF, upon which Collembola feed [10]. Furthermore, there was an interesting interaction between the commercial AM additive and *Sternorrhyncha* where *I. glandulifera* was removed; a negative effect on aboveground *Sternorrhyncha* was observed, suggesting that highly mycorrhizal native plants are unfavourable hosts to these phytophagous insects, while a negative impact was observed for belowground *Sternorrhyncha*, suggesting that higher mycorrhizal colonization improved the aphid build-up on roots [10]. This highlights the important and intricate role of the soil microbiome in mediating interactions between plants and invertebrates and how these processes may be altered by invasives such as *I. glandulifera*. 

## 8. Lesson Number Seven- the Pathway to Becoming an Invasive- Nature or Nurture?

There are a multitude of theories surrounding the pathways to invasion, such as the pre-adaptation hypothesis or the genetic shift hypothesis, and evidence arising from studying *I. glandulifera* suggest that a combination of these theories may provide a more accurate representation of how a species becomes invasive.

### 8.1. Nurture- the Role of the Environment in Enhancing Invasion

The dispersal of *I. glandulifera*, by humans and critically via river catchments, has affected not only the distribution, but the evolution of this species- highlighting the crucial influence of the environment in shaping invasive species [95,96,97]. Indeed, processes relating to river structure, dispersal range and genetic drift appear to contribute towards the structuring of populations of *I. glandulifera* over short temporal scales [95]. Further evidence to support the premise that the invaded environment can shape the genetics, and therefore the success of *I. glandulifera* can be found by studying populations along a latitudinal gradient; a decrease in plant height and biomass was observed with an increase in latitude, alongside an earlier onset of flowering, and this pattern persisted across two generations grown under glasshouse conditions, indicating they are caused by genetic diversity rather than responsive plasticity [65,98]. This genetic de-coupling of vegetative and reproductive traits has enabled *I. glandulifera* to maintain its reproductive vigour while adapting to the environment they encounter, contributing towards its success as an invasive [65].

### 8.2. Genetic Diversity Is Not a Requirement for Successful Invasion

Genetic diversity is not responsible for the success of this invasive, indeed, the genetic diversity of *I. glandulifera* in the invaded range is not as high as in the natural populations in the native foothills of the Himalayas—this is not altogether surprising considering the bottleneck effect of founder populations when invasive species are first introduced to a region, however the extent of the decrease of diversity observed in invasive *I. glandulifera* populations is greater than what is typical for invasive species [65,95,97,99]. Despite this lower than usual genetic diversity, *I. glandulifera* has become a persistent invasive, capable of retaining its genetic diversity at a population level-furthermore *I. glandulifera* is likely to continue to build up higher genetic diversity at a local scale [65]. In fact, despite the reported low genetic diversity, the population genetics are sufficiently diverse to provide resistance to biological control (i.e., *P. glanduliferae*), with some genotypes resistant to two strains of the rust [81,100,101]. Furthermore, a study testing 52 populations of *I. glandulifera* has identified twenty-three haplotypes, with eight of these being unique to the introduced range and only two being found in both invaded and native regions [101].

### 8.3. Nature- the Role of ‘Pre-Adaptation’ in Invasion Success

There is also evidence to support that the very nature of *I. glandulifera* is responsible for its success as an invasive- that it is pre-adapted for invasion, with its inherent characteristics enabling the species to thrive when introduced to more favourable conditions- such as increased nutrients [102]. This theory of pre-adaptation of traits enabling *I. glandulifera* to become invasive when introduced to more favourable conditions is supported by the human facilitated spread of *I. glandulifera* in the native Himalayas (e.g., in Chandrkhani) which has created ‘non-natural’ populations of *I. glandulifera* which display a higher level of performance than ‘natural’ populations [103].

## 9. Conclusions

**Figure 3:**
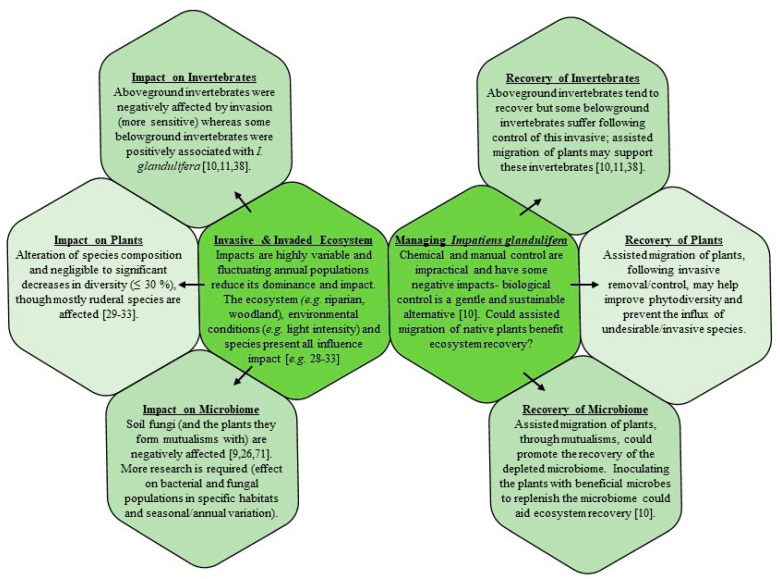
Summary of the impacts of *I. glandulifera* and management strategies. Ecosystem-specific impacts need to be considered when designing control strategies (management or eradication). Both negative and positive impacts need to be considered, alongside potential applications (e.g., bioremediation [45], bioenergy [46], biopharmaceuticals/phytotherapy [47,48,104,105]) some of which could contribute towards funding management strategies. Though it is a ‘backseat driver’ of change in ecosystems, *I. glandulifera* is not as damaging as some other invasive species (such as *Fallopia japonica*) [33,61]. Total eradication may not be feasible, though more recently invaded forests may be easier to manage and removal of plants may still be necessary in heavily invaded areas [10,28,33]. The 2019 update to Regulation (EU) No. 1143/2014 refers to the restoration of the ecosystems damaged by invasion and biological control is a promising management strategy which crucially does not cause further disturbance/damage (physical control can destabilise soil, increasing erosion risk). Assisted migration of native plants may facilitate restoration and help prevent an influx of invasive/weed species [10,51].

Studying *I. glandulifera* has revealed both the potential negative and positive effects of this invasive on the invaded environment. Furthermore, *I. glandulifera* is a good example of how there are potential applications for invasive species, where profits arising from exploiting their traits could be used to aid conservation efforts. Investigating the highly variable impact of *I. glandulifera* on the invaded environment highlights the importance of considering the local, season-specific environmental conditions of microhabitats when completing risk assessments of concerning invasive species. This, alongside insights from experiments examining the efficacy (and environmental impacts) of different control methods, will allow the generation of tailored conservation and management plants to suit specified habitats. Figure 3 summaries the main points from this review, highlighting some of the primary impacts of *I. glandulifera* and insights for management strategies for this invasive, based on the current research presented in this paper. The lessons learnt from studying this invasive have contributed towards our understanding of ecology and evolution, helping to shape future conservation methods and strategies for dealing with invasive species in years to come.

## Data Availability

Not Applicable.
